# When is a target not a target?

**DOI:** 10.7554/eLife.50585

**Published:** 2019-09-12

**Authors:** David C Bersten, Daniel J Peet

**Affiliations:** Department of Molecular and Biological Sciences, School of Biological SciencesUniversity of AdelaideAdelaideAustralia

**Keywords:** hydroxylation, prolyl hydroxylase, oxygenase, Human

## Abstract

Cells rely on prolyl hydroxylase enzymes to sense low levels of oxygen, but they might act on fewer targets than previously thought.

**Related research article** Cockman ME, Lippl K, Tian YM, Pegg HB, Figg WD, Abboud MI, Heilig R, Fischer R, Myllyharju J, Schofield CJ, Ratcliffe PJ. 2019. Lack of activity of recombinant HIF prolyl hydroxylases (PHDs) on reported non-HIF substrates. *eLife* **8**:e46490. doi: 10.7554/eLife.46490

Oxygen is essential for life – just count how many times you need to breathe while reading this article – and is used by virtually every cell in the human body. Most cells are able to sense a diminished oxygen supply (hypoxia) and respond by making changes to cellular metabolism, blood vessel formation and oxygen delivery. For example, physiological hypoxia, such as that encountered in anemia or at high altitudes, induces the production of a hormone called EPO, which causes the body to make more red blood cells to improve oxygen delivery.

Cells must have highly responsive oxygen sensors to regulate these processes, but to date researchers have found only one family of enzymes – the 2-oxoglutarate dioxygenase enzyme family – that is capable of sensing physiological levels of oxygen. These enzymes use molecular oxygen (O_2_), iron ions and 2-oxogluterate (a molecule with the chemical formula C_5_H_6_O_5_) to catalyze the transfer of oxygen onto amino acid or DNA substrates (Islam et al., 2018). They act as both hydroxylases, catalyzing the addition of a hydroxyl group (OH) to substrates, and dioxygenases, using O_2_ as a cosubstrate. Researchers have identified four enzymes from this family that act as oxygen sensors to regulate three transcription factors called HIF1α, HIF2α and HIF3α, which in turn regulate how cells express genes in response to hypoxia. Three of these enzymes are prolyl hydroxylase (PHD) enzymes, and the fourth is called factor inhibiting HIF (Epstein et al., 2001; Ivan et al., 2001; Hewitson et al., 2002; Lando et al., 2002).

The PHD enzymes use molecular oxygen to catalyze the hydroxylation of two proline amino acids in the HIFα proteins. When oxygen levels are normal, the HIFα proteins are hydroxylated, which causes them to be degraded by the cell (Figure 1). However, when oxygen levels decrease, leading to hypoxia, the HIFα proteins are not hydroxylated, so they are not degraded as rapidly. This allows them to migrate to the nucleus and activate the genes responsible for adapting to hypoxia (Kaelin and Ratcliffe, 2008).

**Figure 1.**
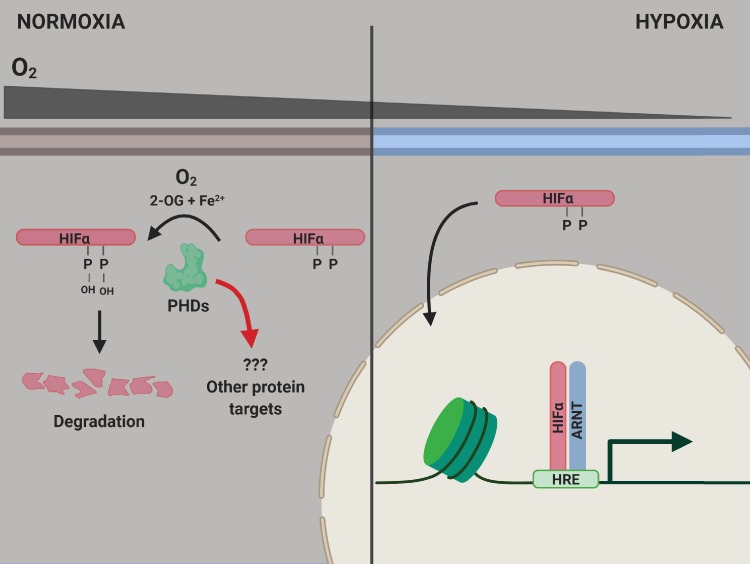
PHD enzymes, HIFα protein and the hypoxia response. When oxygen levels are normal (normoxia, left), a PHD enzyme (green) can use molecular oxygen (O_2_), iron ions (Fe^2+^) and 2-oxogluterate (2-OG) to hydroxylate (ie, add an OH group to) two proline amino acids (P) on a HIFα protein. Hydroxylation destabilizes the HIFα protein, causing it to be degraded by the cell, and the genes involved in the hypoxic response of the cell are not expressed. When oxygen levels are low (hypoxia, right), the PHD enzyme is not able to hydroxylate the HIFα protein, so this protein can migrate into the nucleus and bind to a protein called ARNT. Together, they interact with hypoxia response elements (HREs) in the genome to activate the transcription of hypoxia response genes. ARNT: aryl hydrocarbon receptor nuclear translocator or hypoxic inducible factor-β (HIFβ); HIF: hypoxic inducible transcription factor; PHD: prolyl hydroxylase.

Since the discovery of the PHD enzymes and factor inhibiting HIF, it has been unclear whether these enzymes could hydroxylate targets other than the HIFα proteins. If PHD enzymes hydroxylate other targets it would suggest that additional non-HIF pathways might be involved in the hypoxia response. Previous research efforts have identified many other potential targets for the PHD enzymes, including some with links to physiological responses to hypoxia (Strowitzki et al., 2019). However, many of these studies did not demonstrate that the PHD enzymes were directly catalyzing the hydroxylation of these proteins, raising doubts as to whether these proteins are bona fide targets for the PHD enzymes. Now, in eLife, Matthew Cockman (Francis Crick Institute), Peter Ratcliffe (University of Oxford) and co-workers – including Kerstin Lippl and Ya-Min Tian (both in Oxford) as joint first authors with Cockman, and other researchers from the Crick, Oxford and the University of Oulu – report on a fascinating study that seeks to clarify the situation (Cockman et al., 2019).

Cockman et al. undertook a rigorous array of in vitro biochemical and mass spectrometry experiments using purified enzymes and substrates. While they confirmed that the PHD enzymes robustly catalyze the hydroxylation of proline residues in HIFα proteins, they found no evidence for the hydroxylation of any of the other targets in vitro. Overall, they studied more than 20 different candidate target proteins and 40 potential modification sites. The substrates used in the experiments were short synthetic peptides and full-length recombinant proteins.

These results suggest that the HIFα proteins are the only primary targets of the oxygen-sensing PHD enzymes. If this is the case, then PHD enzymes have a more focused role in hypoxic signaling than previously thought. This is important for predicting the consequences of manipulating PHD activity for therapeutic purposes. However, while these well-controlled, designed and executed biochemical experiments show that targets other than HIFα proteins cannot be efficiently hydroxylated in vitro, they do not preclude the modification of these targets in vivo. This is because living cells might contain additional cofactors that the PHD enzymes need to hydroxylate non-HIFα targets. Furthermore, modulating the activity of the PHD enzymes affects HIF-independent processes, indirectly pointing to potential non-HIF targets. (Strowitzki et al., 2019).

If in vivo experiments confirm that the HIFα proteins are the only primary targets of the PHD enzymes, as Cockman et al. suggest, this would make these enzymes central to of one of the most specialized sensing and control systems in the cell.
